# Supplementary material for: Structural and biochemical comparison of the FLVCR and CTL membrane protein families in eukaryotes
Source: Life Sci Alliance. 2026 May 11;9(7):e202503583. doi: 10.26508/lsa.202503583 (PMC13160678; doi:10.26508/lsa.202503583)
Supplement: Supplementary file 1 [file LSA-2025-03583_TableS1.docx]

**Table S1:** Summary of cryo-EM date collection, refinement and validation

| Protein | **FLVCR2** | **hCTL1** | **hCTL1** |
| --- | --- | --- | --- |
| Sample composition | DDM detergent, pH 7.5 | Peptidisc, pH 7.4 | LMNG detergent, pH 7.4 |
| **Data collection** | | | |
| Microscope | FEI Titan Krios | | |
| EM facility | EMBION | | |
| Voltage (kV) | 300 | | |
| Detector | Gatan K3 | | |
| Magnification (times) | 130,000 | | |
| Raw pixel size (Å) | 0.647 | | |
| Dose (e^-^/Å^2^) | 60 | | |
| Defocus range (μm) | 0.6 – 1.8 | | |
| Number of exposures^#^ | 6,605 | 6,758 | 6,758 |
| **Reconstruction** | | | |
| Software | cryoSPARC (v.4.4.1) | | |
| Final number of particles from images | 271,101 | 64,034 | 115,320 |
| Symmetry imposed | C1 | C1 | C1 |
| Map resolution (Å)* | 3.39 | 3.3 | 3.3 |
| **Model building** | | | |
| Software | ISOLDE and COOT | Not applicable | ISOLDE and COOT |
| Starting model | AlphaFold 2 prediction |  | AlphaFold 2 prediction |
| **Refinement** | | | |
| Software | Phenix |  | Phenix |
| Model composition  Non-hydrogen atoms  Total atoms  Residues  Ligands  Waters | 3137  6381  402  CHT:1  0 | Not applicable | 2889  5787  362  0  0 |
| RMSD  Bond lengths (Å)  Bond angles (degrees) | 0.003  0.511 |  | 0.002  0.442 |
| **Validation** | | | |
| MolProbity score | 1.45 | Not applicable | 1.18 |
| Clashscore | 5.65 |  | 3.89 |
| Model-to-map-fit | 0.7612 |  | 0.7512 |
| Rotamer outliers (%) | 0.89 |  | 0.94 |
| Rama-Z score (RMSD)  Whole/Helix/Loop | 2.41/2.15/-1.34 |  | 2.47/1.88/0.74 |
| CaBLAM outliers (%) | 0.51 |  | 0.00 |
| Ramachandran statistics (%)  Outliers  Allowed  Favoured | 0.00  2.76  97.24 |  | 0.00  2.00  98.00 |
| Accession number | PDB: 9QU4; EMD-53371 | EMD-50251 | PDB: 9QU3; EMD-50252 |

^#^After curation. *Gold-standard FSC at 0.143.
