# Supplementary material for: Structural and biochemical comparison of the FLVCR and CTL membrane protein families in eukaryotes
Source: Life Sci Alliance. 2026 May 11;9(7):e202503583. doi: 10.26508/lsa.202503583 (PMC13160678; doi:10.26508/lsa.202503583)
Supplement: Supplementary file 2 [file LSA-2025-03583_TableS2.docx]

**Table S2:** X-ray crystallography data collection and refinement statistics.

| Dataset | **PNS1 native** |
| --- | --- |
| **Data collection** | |
| Space group | P22_1_2_1_ |
| Cell dimensions  a; b; c (Å)  α; β; χ (°) | 67.4; 91.3; 108.4  90; 90; 90 |
| Monomers per asymmetric unit | 1 |
| Wavelength (Å) | 0.9764 |
| Number of reflections measured  Total  Unique | 18374 |
| Resolution range (Å) | 2.7 - 48.5 (2.79 - 2.94)^¤^ |
| R_meas_ | 0.145 (2.399) |
| Mean I/sigma(I) | 8.8 (0.9) |
| CC½ | 0.998 (0.413) |
| Multiplicity | 6.4 (6.7) |
| Completeness (%) | 99 (100) |
| Wilson B-factor (Å) | 81 |
| **Refinement** | |
| Number of reflections (work/free)  R_work_ (%)  R_free_ (%) | 18314/918  23.2  28.4 |
| Number of atoms  Protein  Ligand  Solvent | 3369  -  12 |
| Average B-factor  Macromolecules  Ligands  Solvent | 88.2  -  81.6 |
| RMSD  Bond lengths (Å)  Bond angles (°) | 0.0067  0.86 |
| Ramachandran statistics (%)  Favoured  Allowed  Disallowed | 98.55  1.45  0.00 |
| Clashscore | 7.24 |
| Number of TLS groups | 2 |
| Accession number | PDB: 9F63 |

^¤^Highest resolution shell is shown in parenthesis.
